# Prevalence and Trends in Prepregnancy Normal Weight — 48 States, New York City, and District of Columbia, 2011–2015

**DOI:** 10.15585/mmwr.mm665152a3

**Published:** 2018-01-05

**Authors:** Nicholas P. Deputy, Bhanuja Dub, Andrea J. Sharma

**Affiliations:** ^1^Division of Reproductive Health, National Center for Chronic Disease Prevention and Health Promotion, CDC; ^2^Oak Ridge Institute for Science and Education Fellowship, U.S. Department of Energy; ^3^Rollins School of Public Health, Emory University, Atlanta, Georgia.

Women who enter pregnancy at a weight above or below normal weight, defined as a body mass index (BMI) of 18.5–24.9 (calculated as weight in kg/height in m^2^), are more likely to experience adverse pregnancy outcomes and to have infants who experience adverse health outcomes. For example, prepregnancy underweight (BMI <18.5) increases the risk for small-for-gestational-age births, whereas prepregnancy overweight (BMI 25.0–29.9) and obesity (BMI ≥30.0) increase risks for cesarean delivery, large-for-gestational-age births, and childhood obesity (*1*). Given these outcomes, *Healthy People 2020* includes an objective to increase the proportion of women entering pregnancy with a normal weight from 52.5% in 2007 to 57.8% by 2020.* Because recent trends in prepregnancy normal weight have not been reported, CDC examined 2011–2015 National Vital Statistics System (NVSS) natality data, which included prepregnancy BMI. In 2015, for 48 states, the District of Columbia (DC), and New York City (NYC) combined, the prevalence of prepregnancy normal weight was 45.0%; prevalence ranged from 37.7% in Mississippi to 52.2% in DC. Among 38 jurisdictions with prepregnancy BMI data during 2011–2015, normal weight prevalence declined from 47.3% to 45.1%; declines were observed in all jurisdictions but were statistically significant for 27 jurisdictions after standardizing to the 2011 national maternal age and race/ethnicity distribution. Screening women’s BMI during routine clinical care provides opportunities to promote normal weight before entering pregnancy.

NVSS collects demographic and health information for live births in 50 states^†^ and DC via the U.S. Standard Certificate of Live Birth (birth certificate), which was revised in 2003 to include maternal height and prepregnancy weight. Height and prepregnancy weight are self-reported or abstracted from medical records^§^ and are used by NVSS to calculate prepregnancy BMI. The revised birth certificate was used in 36 states, DC, and NYC by 2011 and was used in 48 states, DC, and NYC by 2015 (representing 83% and 97% of all live births in 2011 and 2015, respectively).^¶^ Births to U.S. resident mothers in states adopting the revised birth certificate by January 1 of each year were eligible for analyses (17,906,182 mothers, representing 90% of all U.S. births during 2011–2015).** From these records, those with missing BMI (732,052) were excluded, resulting in 17,174,130 records for analysis (96% of births eligible for this analysis).

Prepregnancy BMI was categorized as underweight (<18.5), normal weight (18.5–24.9), overweight (25.0–29.9), or obese (≥30.0); for some analyses, obesity was categorized as class I (BMI = 30.0–34.9), class II (35.0–39.9), or class III (≥40.0). Overall and jurisdiction-specific prevalences for each prepregnancy BMI category were estimated. Overall and jurisdiction-specific trends were estimated as the percentage-point difference in prepregnancy normal weight prevalence from 2011 to 2015 for 38 jurisdictions with available data; overall trends for each prepregnancy BMI category were also estimated as the percentage change from 2011 to 2015. Because prepregnancy BMI increases with maternal age and varies by maternal race/ethnicity (*2*), jurisdiction-specific differences were estimated after directly standardizing each year to the race/ethnicity and age distribution^††^ of 2011 U.S. resident mothers to facilitate comparisons. Standardized, jurisdiction-specific differences were evaluated using the z-statistic; p<0.05 was considered statistically significant.

For 48 states, DC, and NYC in 2015, the overall prevalence of prepregnancy normal weight was 45.0%; prevalences ranged from 37.7% in Mississippi to 52.2% in DC (Table 1). Among 38 jurisdictions with prepregnancy BMI data from 2011 to 2015, prevalence of normal weight declined from 47.3% to 45.1%; after standardization, this represented a 1.9 percentage-point decline (p<0.05). Declines in prepregnancy normal weight were observed in all 38 jurisdictions, but were statistically significant in 27 jurisdictions; declines ranged from 1.0 percentage point (p = 0.01) in Wisconsin to 3.5 percentage points (p<0.001) in Delaware over the 5-year period (Table 1).

**TABLE 1:** Prevalence of prepregnancy normal weight* among women with a live birth, by jurisdiction and year — 48 states,**^†^** District of Columbia, and New York City, 2011–2015

Jurisdiction	No. of live births					% of women with prepregnancy normal weight^§^					Percentage-point difference in standardized^¶^ prevalence from 2011 to 2015
	2011	2012	2013	2014	2015	2011	2012	2013	2014	2015	
**Alabama**	—**	**—**	**—**	**57,563**	**58,312**	**—**	**—**	**—**	**42.3**	**40.9**	**—**
Alaska	—	—	10,871	11,101	10,956	—	—	46.4	45.9	46.2	—
Arizona	—	—	—	86,351	84,960	—	—	—	44.9	43.9	—
Arkansas	—	—	—	37,459	37,599	—	—	—	42.1	42.9	—
California	474,514	477,348	470,386	481,030	473,927	48.5	48.2	47.6	47.5	46.7	-1.7^††^
Colorado	63,266	63,372	63,340	63,909	64,528	52.3	51.1	50.5	50.6	49.5	-2.7^††^
Delaware	11,059	10,916	10,696	10,849	11,071	45.1	45.1	43.8	42.7	41.0	-3.5^††^
District of Columbia	8,050	8,597	8,608	9,022	9,240	52.7	52.4	52.6	53.0	52.2	-1.8
Florida	202,005	201,549	202,173	206,871	211,232	48.3	48.2	47.3	47.1	46.5	-1.7^††^
Georgia	102,287	110,951	109,530	116,260	121,378	42.6	42.3	41.5	42.3	42.1	-0.2
Hawaii	—	—	—	17,661	17,653	—	—	—	48.5	47.8	—
Idaho	22,232	22,883	22,299	22,819	22,703	50.1	49.3	48.8	48.4	47.4	-2.8^††^
Illinois	156,300	153,521	150,347	152,685	150,222	46.0	45.5	44.5	44.4	42.8	-2.9^††^
Indiana	82,794	82,545	82,442	83,736	83,727	45.0	44.4	43.2	43.1	42.0	-2.6^††^
Iowa	38,061	38,555	38,964	39,512	39,281	46.5	46.0	45.1	44.8	44.6	-1.5^††^
Kansas	38,588	39,479	38,095	38,676	38,999	46.8	46.3	45.9	44.5	44.0	-2.7^††^
Kentucky	54,413	54,873	54,706	55,653	55,397	43.7	43.0	42.2	42.1	41.1	-2.2^††^
Louisiana	59,214	60,165	60,920	62,428	62,191	43.8	43.1	43.0	42.5	41.3	-1.9^††^
Maine	—	—	—	12,585	12,562	—	—	—	43.1	41.7	—
Maryland	69,775	70,093	69,045	71,388	71,406	46.5	46.0	45.8	45.4	44.3	-2.1^††^
Massachusetts	—	68,218	66,589	67,812	68,945	—	52.5	52.4	51.9	51.0	—
Michigan	109,157	108,065	108,462	110,080	109,542	45.1	44.6	44.0	43.3	42.4	-2.5^††^
Minnesota	—	66,583	67,735	68,472	67,775	—	45.5	45.5	44.9	43.8	—
Mississippi	—	—	38,056	38,554	38,232	—	—	39.7	39.4	37.7	—
Missouri	74,491	74,038	73,978	74,352	74,121	47.3	47.0	46.5	46.0	45.3	-1.8^††^
Montana	11,761	11,652	11,963	12,241	12,458	49.0	48.8	48.5	48.0	46.5	-3.5
Nebraska	25,465	25,710	25,859	26,531	26,434	48.4	48.4	47.1	46.9	46.3	-2.1^††^
Nevada	34,793	34,521	34,636	35,288	35,694	48.8	48.9	47.9	47.2	46.4	-1.8^††^
New Hampshire	11,820	11,391	11,590	11,649	11,844	50.1	49.5	48.9	47.7	47.4	-2.4
New Mexico	25,390	25,447	25,028	24,666	24,899	43.2	44.1	44.3	43.6	42.0	-0.1
New York^§§^	114,593	114,215	113,392	111,635	112,131	46.7	46.3	46.0	45.1	44.3	-2.2^††^
New York City	117,787	118,093	115,251	116,281	115,814	53.2	53.5	53.0	52.2	52.1	-1.3^††^
North Carolina	116,970	116,249	116,489	118,550	117,841	46.4	45.8	45.1	45.1	44.5	-1.8^††^
North Dakota	9,382	9,948	10,364	11,115	11,155	41.0	41.0	42.4	41.8	40.2	-2.5
Ohio	130,723	131,056	131,785	135,214	135,442	46.9	46.7	46.2	45.7	44.8	-2.0^††^
Oklahoma	50,824	51,139	51,676	52,323	52,024	45.2	44.5	43.5	42.5	41.7	-3.2^††^
Oregon	44,311	43,917	43,909	44,675	45,098	48.5	47.9	48.2	47.7	47.0	-1.3^††^
Pennsylvania	130,461	128,323	126,663	133,108	130,973	49.2	48.8	48.4	48.4	47.1	-2.0^††^
Rhode Island	—	—	—	—	10,431	—	—	—	—	48.2	—
South Carolina	56,023	55,267	55,576	56,919	57,333	42.5	42.9	41.9	41.8	41.3	-0.6
South Dakota	11,675	11,954	12,094	12,136	12,194	47.8	48.6	47.0	47.8	46.5	-1.0
Tennessee	76,586	77,402	77,400	79,112	78,735	46.5	46.5	45.9	45.2	44.7	-1.6^††^
Texas	374,890	380,229	385,536	396,957	401,330	47.4	46.8	46.0	45.3	44.5	-1.9^††^
Utah	49,951	50,670	50,181	50,473	50,239	54.9	54.0	53.7	53.1	51.9	-2.4^††^
Vermont	5,957	5,927	5,900	6,053	5,818	49.4	49.2	47.9	47.6	46.7	−6.0
Virginia	—	—	74,145	77,879	91,400	—	—	48.4	48.1	45.4	—
Washington	81,676	83,051	81,723	83,821	84,917	46.4	45.9	45.5	45.3	45.9	-0.4
West Virginia	—	—	—	19,709	19,489	—	—	—	42.4	40.1	—
Wisconsin	66,647	66,342	65,556	65,915	65,727	43.1	43.2	42.9	42.3	41.7	-1.0^††^
Wyoming	7,278	7,448	7,532	7,609	7,703	50.1	49.9	50.3	50.2	49.0	-3.1
**38 jurisdictions with BMI data from 2011 to 2015**	**3,121,169**	**3,136,901**	**3,124,094**	**3,191,541**	**3,194,768**	**47.3**	**46.9**	**46.3**	**45.9**	**45.1**	**-1.9^††^**
**All jurisdictions with available data**	**3,121,169**	**3,271,702**	**3,381,490**	**3,686,687**	**3,713,082**	**47.3**	**47.0**	**46.4**	**45.9**	**45.0**	**-2.1^††^**

**Abbreviation**: BMI = body mass index (kg/m^2^).

* BMI = 18.5–24.9.

^†^ Connecticut and New Jersey did not use the revised birth certificate by January 1, 2015.

^§^ Crude prevalence.

^¶^ Standardized to 2011 race/ethnicity and age distribution.

** Revised birth certificate data not available for that jurisdiction during that year.

^††^ Statistically significant (p<0.05) decrease in mean prevalence standardized to the 2011 maternal age and race/ethnicity distribution.

^§§^ Natality data from New York City are reported separately and are not included in New York state estimates.

Corresponding with the decline in prepregnancy normal weight prevalence during 2011–2015, the entire BMI distribution shifted toward a higher BMI (Figure). Specifically, there was an 8% decrease in the prepregnancy underweight prevalence, while there were 2% and 8% increases in overweight and obesity, respectively. Notably, class III obesity prevalence increased more rapidly than did class I or class II obesity (increase of 14% [class III], compared with 10% [class II] and 6% [class I]).

**FIGURE:**
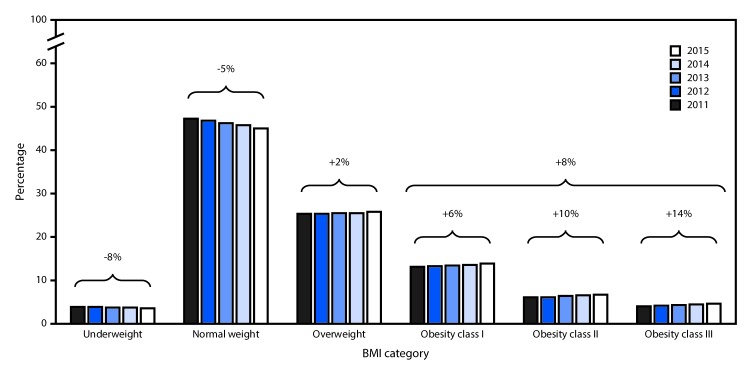
Prevalences and relative changes in prepregnancy BMI categories* among women with a live birth — 36 states, District of Columbia, and New York City,**^†^** 2011–2015 **Abbreviation**: BMI = body mass index (kg/m^2^). * Prepregnancy BMI was categorized as underweight (BMI <18.5), normal weight (BMI 18.5–24.9), overweight (BMI 25.0–29.9), obesity class I (BMI 30.0–34.9), obesity class II (BMI 35.0–39.9), and obesity class III (BMI ≥40.0). ^^†^^ Data are from 38 jurisdictions that utilized the revised birth certificate by January 1, 2011 and, thus, had prepregnancy BMI data during 2011–2015. Jurisdictions included are California, Colorado, Delaware, District of Columbia, Florida, Georgia, Idaho, Illinois, Indiana, Iowa, Kansas, Kentucky, Louisiana, Maryland, Michigan, Missouri, Montana, Nebraska, Nevada, New Hampshire, New Mexico, New York, New York City, North Carolina, North Dakota, Ohio, Oklahoma, Oregon, Pennsylvania, South Carolina, South Dakota, Tennessee, Texas, Utah, Vermont, Washington, Wisconsin, and Wyoming (natality data from New York City are reported separately and are not included in New York estimates).

In 2015, jurisdictions with the highest prepregnancy normal weight prevalence (DC, Massachusetts, NYC, and Utah) had the lowest obesity prevalence, whereas jurisdictions with lowest prepregnancy normal weight prevalence (Mississippi and West Virginia) had the highest obesity prevalence (Table 2). Although NYC had a relatively high prevalence of prepregnancy normal weight, it also had the highest prevalence of underweight. Notably, some states exhibited a double burden of higher prevalences of prepregnancy underweight and obesity (Arkansas, Kentucky, and West Virginia).

**TABLE 2:** Prevalence of prepregnancy BMI categories* among women with a live birth, by jurisdiction — 48 states,^†^ District of Columbia, and New York City, 2015

Jurisdiction	% Underweight	% Normal weight	% Overweight	% Obese
Alabama	3.9	40.9	24.8	30.4
Alaska	2.4	46.2	25.4	26.0
Arizona	3.8	43.9	26.1	26.1
Arkansas	4.0	42.9	23.7	29.5
California	3.7	46.7	26.4	23.2
Colorado	3.5	49.5	26.1	20.9
Delaware	3.3	41.0	27.7	28.0
District of Columbia	4.4	52.2	23.4	19.9
Florida	4.2	46.5	26.1	23.3
Georgia	3.8	42.1	25.9	28.3
Hawaii	4.2	47.8	25.2	22.8
Idaho	3.2	47.4	25.2	24.2
Illinois	3.1	42.8	26.8	27.3
Indiana	3.5	42.0	25.8	28.7
Iowa	2.9	44.6	25.7	26.8
Kansas	3.2	44.0	26.4	26.4
Kentucky	4.1	41.1	24.8	30.0
Louisiana	3.8	41.3	25.1	29.9
Maine	2.0	41.7	26.2	30.1
Maryland	3.1	44.3	26.8	25.7
Massachusetts	3.5	51.0	25.3	20.3
Michigan	3.2	42.4	25.9	28.6
Minnesota	2.2	43.8	27.7	26.3
Mississippi	3.8	37.7	25.0	33.5
Missouri	3.8	45.3	24.5	26.4
Montana	3.3	46.5	25.5	24.7
Nebraska	2.9	46.3	26.0	24.7
Nevada	4.4	46.4	25.4	23.8
New Hampshire	2.8	47.4	25.9	23.9
New Mexico	3.9	42.0	26.8	27.2
New York^§^	2.9	44.3	27.0	25.8
New York City	5.4	52.1	24.8	17.8
North Carolina	3.8	44.5	25.2	26.6
North Dakota	2.3	40.2	27.8	29.7
Ohio	3.7	44.8	24.6	26.9
Oklahoma	3.8	41.7	25.7	28.8
Oregon	3.1	47.0	25.0	24.9
Pennsylvania	3.6	47.1	24.6	24.6
Rhode Island	2.8	48.2	26.6	22.4
South Carolina	3.7	41.3	25.3	29.7
South Dakota	3.0	46.5	25.7	24.9
Tennessee	4.4	44.7	24.4	26.4
Texas	3.6	44.5	26.4	25.6
Utah	4.1	51.9	23.5	20.5
Vermont	2.8	46.7	24.3	26.1
Virginia	3.4	45.4	26.4	24.7
Washington	3.1	45.9	26.0	25.0
West Virginia	4.7	40.1	23.9	31.3
Wisconsin	2.2	41.7	26.3	29.8
Wyoming	3.4	49.0	24.7	22.9
**Total**	**3.6**	**45.0**	**25.8**	**25.6**

**Abbreviation**: BMI = body mass index (kg/m^2^).

* Prepregnancy BMI was categorized as underweight (BMI <18.5), normal weight (BMI 18.5–24.9), overweight (BMI 25.0–29.9), and obese (BMI ≥30.0).

^†^ Connecticut and New Jersey did not use the revised birth certificate by January 1, 2015.

^§^ Natality data from New York City are reported separately and are not included in New York state estimate.

## Discussion

Among the 48 states, DC, and NYC that implemented the revised birth certificate, the overall prevalence of prepregnancy normal weight in 2015 was 45.0%. Among 38 jurisdictions with prepregnancy BMI data from 2011 to 2015, the prevalence of prepregnancy normal weight declined by 5%, whereas the prevalence of overweight increased by 2%, and the prevalence of obesity (all classes) increased by 8%; taken together, these results suggest movement away from the *Healthy People 2020* target for prepregnancy normal weight.

Trends from this analysis extend previous findings and demonstrate continued declines in prepregnancy normal weight prevalence. Data from 20 states participating in the Pregnancy Risk Assessment Monitoring System, a multistate representative surveillance system, found prevalence of prepregnancy normal weight declined from 54.5% in 2003 to 51.5% in 2009 (*3*). Data from the National Health and Nutrition Examination Survey indicate prevalence of normal weight also declined among nonpregnant women aged 20–34 years, from 42.5% in 1999–2002 to 38.1% in 2011–2014; similar declines were observed for women aged 35–44 years (*4*). The declining prevalence of prepregnancy normal weight is concerning because of adverse outcomes associated with entering pregnancy outside of normal weight. For example, prepregnancy underweight increases risks for preterm delivery and small-for-gestational-age births, whereas prepregnancy overweight and obesity increase risks for gestational diabetes mellitus and childhood obesity (*1*). Moreover, obesity during pregnancy has been associated with increased health care service utilization, including longer hospital stays during delivery (*5*). Before pregnancy, obesity among women of reproductive age is associated with reduced fertility and potentially increased use of fertility treatments (*6*).

Preconception care is the provision of medical care and interventions that promote optimal health for reproductive-age women and also promote optimal pregnancy outcomes should a pregnancy occur (*7*). Weight-related screening, counseling, and referral for treatment services are some of the components of preconception care (*7*,*8*). The U.S. Preventive Services Task Force recommends that clinicians assess BMI to screen all adults for obesity and offer patients with obesity intensive, multicomponent behavioral interventions or refer patients for these interventions.^§§^ The American College of Obstetricians and Gynecologists (ACOG) recommends BMI screening during routine well-woman visits^¶¶^ and recently released an online toolkit^†††^ to facilitate BMI screening and referral for treatment. The toolkit includes an obesity assessment algorithm, counseling methods, treatment options, referral resources, and a coding guide to facilitate reimbursement. For women with underweight BMI, ACOG recommends that clinicians counsel patients about adverse pregnancy outcomes associated with underweight and assess for disordered eating habits (*8*). Reports indicate prevalence of prepregnancy underweight is highest among women aged <20 years (*2*), possibly because adult BMI criteria are applied to pregnancies among adults and adolescents (*9*); this categorizes more adolescents as underweight than the pediatric growth charts and results in higher recommended pregnancy weight gain, which has been found to improve pregnancy outcomes among adolescents (*9*).

The findings in this report are subject to at least three limitations. First, height and prepregnancy weight on the birth certificate are self-reported or abstracted from medical records, which might result in misclassification of BMI category. Second, results of this analysis are not directly comparable to *Healthy People 2020* targets for prepregnancy normal weight because these targets were developed using surveillance data from 29 states that exclusively rely on height and prepregnancy weight self-reported 2–7 months postpartum; thus, these targets might differ from those developed using birth certificate data. Notably, the revised birth certificate is a census of all births, which will allow for ongoing monitoring of prepregnancy weight in all states. Finally, data were not available from all states for trend analyses; thus, results do not represent the entire U.S. population of women giving birth.

In 2015, the nearly national prevalence of prepregnancy normal weight was 45.0% and prevalence declined from 2011 to 2015 in most jurisdictions, suggesting movement away from the *Healthy People 2020* objective to increase the prevalence of prepregnancy normal weight. For all women of reproductive age, BMI screening during routine clinical visits provides opportunities to address underweight or obesity, promote normal weight upon entering pregnancy, and ultimately help optimize maternal and child health outcomes.

**Summary**

**What is already known about this topic?**

Entering pregnancy outside a normal weight (body mass index [BMI] of 18.5–24.9 kg/m^2^) is associated with adverse maternal and infant health outcomes; given these outcomes, *Healthy People 2020* includes an objective to increase the proportion of women entering pregnancy with normal weight. Recent trends in national or jurisdiction-specific prevalence of prepregnancy normal weight have not been reported.

**What is added by this report?**

Using data from the revised birth certificate for 48 states, the District of Columbia (DC), and New York City (NYC), this analysis found that the overall prevalence of prepregnancy normal weight was 45.0% in 2015; prevalence ranged from 37.7% in Mississippi to 52.2% in DC. Among 36 states, DC, and NYC with available prepregnancy BMI data from 2011 to 2015, prevalence of normal weight declined from 47.3% to 45.1%; declines were observed in all jurisdictions but were statistically significant among 27 after standardizing to the 2011 national maternal age and race/ethnicity distribution.

**What are the implications for public health practice?**

Overall and among most jurisdictions examined, the prevalence of prepregnancy normal weight is decreasing; this suggests movement away from the *Healthy People 2020* objective for prepregnancy normal weight. For women of reproductive age, BMI screening during routine clinical visits provides opportunities to address underweight or obesity, promote normal weight upon entering pregnancy, and ultimately help optimize maternal and child health outcomes.

## Footnotes

* *Healthy People 2020* Maternal, Infant and Child Health (MICH) Objective 16.5 for healthy prepregnancy weight (defined as normal weight BMI) was developed using state-specific surveillance data that rely on self-reported height and prepregnancy weight reported approximately 2–7 months postpartum. Data from 28 states participating in the Pregnancy Risk Assessment Monitoring System and data from California’s Maternal and Infant Health Assessment survey contributed to the development of this objective. https://www.healthypeople.gov/2020/topics-objectives/topic/Maternal-Infant-and-Child-Health/objectives.

† Natality data from New York City are reported separately from those for the state of New York and are not included in New York estimates.

§ Per National Center for Health Statistics guidance for completing the 2003 revision of the U.S. Standard Certificate of Live Birth, the preferred source for prepregnancy weight and height is self-report by the mother around the time of delivery, which is recorded on the Mother’s Worksheet (https://www.cdc.gov/nchs/data/dvs/momswkstf_improv.pdf). Maternal height and prepregnancy weight recorded in the mother’s prenatal care record may be used as an alternative source. https://www.cdc.gov/nchs/data/dvs/birth_edit_specifications.pdf.

¶ Connecticut and New Jersey did not use the revised birth certificate by January 1, 2015.

** For each year from 2011 to 2015, the distributions of maternal race/ethnicity and age were not meaningfully different for women residing in states that used the revised birth certificate compared with the entire population of women giving birth in the United States. Additional information can be found in the Birth Data File User’s Guide for each year. https://www.cdc.gov/nchs/data_access/Vitalstatsonline.htm.

†† Race/ethnicity was classified as Hispanic, non-Hispanic white, black, American Indian/Alaska Native, and Asian/Pacific Islander. Age was categorized into the following age groups: <19, 20–24, 25–29, 30–34, and >35 years.

§§ https://www.uspreventiveservicestaskforce.org/Page/Document/RecommendationStatementFinal/obesity-in-adults-screening-and-management.

¶¶ https://www.acog.org/wellwoman.

†††
https://www.acog.org/About-ACOG/ACOG-Departments/Toolkits-for-Health-Care-Providers/Obesity-Toolkit .
